# Performance evaluation of unified medical language system^®^'s synonyms expansion to query PubMed

**DOI:** 10.1186/1472-6947-12-12

**Published:** 2012-02-29

**Authors:** Nicolas Griffon, Wiem Chebil, Laetitia Rollin, Gaetan Kerdelhue, Benoit Thirion, Jean-François Gehanno, Stéfan Jacques Darmoni

**Affiliations:** 1 CISMeF, Rouen University Hospital, Cour Leschevin, Porte 21, 3ème étage. 1 rue de Germont, 76031 Rouen Cedex, France; 2 TIBS, Rouen University, LITIS EA 4108, Rouen, France; 3 Institute of Management, University of Sousse, Sousse, Tunisia

## Abstract

**Background:**

PubMed is the main access to medical literature on the Internet. In order to enhance the performance of its information retrieval tools, primarily non-indexed citations, the authors propose a method: expanding users' queries using Unified Medical Language System' (UMLS) synonyms i.e. all the terms gathered under one unique Concept Unique Identifier.

**Methods:**

This method was evaluated using queries constructed to emphasize the differences between this new method and the current PubMed automatic term mapping. Four experts assessed citation relevance.

**Results:**

Using UMLS, we were able to retrieve new citations in 45.5% of queries, which implies a small increase in recall. The new strategy led to a heterogeneous 23.7% mean increase in non-indexed citation retrieved. Of these, 82% have been published less than 4 months earlier. The overall mean precision was 48.4% but differed according to the evaluators, ranging from 36.7% to 88.1% (Inter rater agreement was poor: kappa = 0.34).

**Conclusions:**

This study highlights the need for specific search tools for each type of user and use-cases. The proposed strategy may be useful to retrieve recent scientific advancement.

## Background

The most important tool to access the medical literature is the PubMed search engine, which allows access to more than 20 millions of biomedical citations. The major part of these citations comes from the MEDLINE bibliographic database, which uses the MeSH thesaurus for indexing [1]. Other citations, i.e. OLDMEDLINE, out of scope or recent citations, are not indexed at the time of user query [2]. The most comprehensive way to find citations in MEDLINE is to use the MeSH thesaurus.

Because one third of Medline queries are performed by members of the general public [3] and furthermore because most health professionals [4] are not aware of this thesaurus, they run free-text queries, as they do when using Google™. This allows searching the entire PubMed collection but does not at all exploit the indexing work produced by National Library of Medicine (NLM) building the MeSH thesaurus and indexing millions of citations. Consequently, the US National Center of Biotechnology Information has developed several techniques (Automatic Term Mapping (ATM)) to map end-user queries to the MeSH thesaurus and other search field descriptors (e.g. author's name, publication's name, etc.) [5]. The first ATM aim is to improve information retrieval in structured information: searching indexes (mostly MeSH terms used to index citations in MEDLINE) instead of only the free text. Almost nothing is done to enhance the search for recent citations (not indexed). This is a limiting factor because 1) these citations contain the most recent scientific discoveries and 2) they are the first returned by PubMed, which displays recent articles first, by default.

Although PubMed ATM query is continuously improved, a recent review [6] has counted 28 different entities that have devoted themselves to develop Web tools for helping users to quickly and effectively search and retrieve relevant publications on MEDLINE. This highlights the need for alternative ways of searching the medical literature. Thirion et al. [7] have shown that it is possible to improve ATM's performance, mainly in precision (tools available with the Doc'CISMeF search engine http://www.cismef.org) using MeSH synonyms. The aim of this paper was to propose an extension to this previous optimization, using Unified Medical Language System^® ^(UMLS) synonyms, and to assess its performance.

## Methods

### MeSH & UMLS

The MeSH is the terminology, covering the whole area of medicine, used by the NLM for indexing MEDLINE citations. Each MeSH descriptor is named by a preferred term and may have some entry terms or synonyms, e.g. "myocardial infarction" is the preferred term designating the same MeSH descriptor rather than "myocardial infarct", "infarct, myocardial", etc. which are entry terms, or synonyms.

The UMLS contains a metathesaurus gathering many health terminologies/ontologies (T/O), like MeSH. For each T/O, each term is assigned to one or more concepts in UMLS. We defined as UMLS synonyms all the different terms from different T/O gathered under the same UMLS concept (same Concept Unique Identifier), e.g. "myocardial infarction" from the MeSH, "myocardial infarction" from the WHO Adverse Reaction Terminology (WHO-ART) and "heart attack" from the WHO-ART, etc. are UMLS synonyms as they are within the same UMLS concept.

### Queries

In April 2011, when the ATM was used to match some query terms with a MeSH term, the resulting modified query was different if the query terms matched with the preferred term or an entry term or a UMLS' synonym [5]. If it was the preferred term, the resulting modified query was: *q1 = "preferred term"[MeSH term] OR "preferred term"[all fields] OR ("word_1 _of preferred term"[All Fields] AND "word_2 _of preferred term"[All Fields] AND *etc.) (Table 1). If it was an entry term or a UMLS' synonym, the resulting modified query was: *q2 = "preferred term"[MeSH term] OR "preferred term"[all fields] OR ("word_1 _of preferred term"[All Fields] AND "word_2 _of preferred term"[All Fields] AND *etc.) *OR "entry term"[All Fields] OR ("word_1 _of entry term"[All Fields] AND "word_2 _of entry term"[All Fields] AND *etc.) (Table 1).

**Table 1:** Examples of query

Query		Query syntax
	
	Preferred term	Entry term or UMLS' synonym
**User**	**Myocardial infarction**	**Myocardial infarct**

q1	"myocardial infarction"[MeSH Terms] OR ("myocardial"[All Fields] AND "infarction"[All Fields]) OR "myocardial infarction"[All Fields]	

q2		"myocardial infarction"[MeSH Terms] OR ("myocardial"[All Fields] AND "infarction"[All Fields]) OR "myocardial infarction"[All Fields] OR ("myocardial"[All Fields] AND "infarct"[All Fields]) OR "myocardial infarct"[All Fields]

q3	"myocardial infarction"[MeSH Terms] OR (("infarct, myocardial"[TIAB] OR "infarction, myocardial"[TIAB] OR "myocardial infarcts"[TIAB] OR "myocardial infarct"[TIAB] OR "myocardial infarction"[TIAB] OR "infarcts, myocardial"[TIAB] OR "myocardial infarctions"[TIAB] OR "infarctions, myocardial"[TIAB]) NOT MEDLINE[SB])	

q4	"myocardial infarction"[MeSH Terms] OR (("infarct, myocardial"[TIAB] OR "heart attack"[TIAB] OR "infarction, myocardial"[TIAB] OR "myocardial infarcts"[TIAB] OR "myocardial infarct"[TIAB] OR "myocardial infarction"[TIAB] OR "myocardial infarction, nos"[TIAB] OR "infarcts, myocardial"[TIAB] OR "myocardial infarctions"[TIAB] OR "infarctions, myocardial"[TIAB]) NOT (MEDLINE[SB] OR OldMedline[SB]))	

q5	q4 NOT q3	

TIAB: title or abstract; SB: Subset; UMLS' synonyms are underlined

However, these queries were not the same compared to Thirion et al.'s strategies [7], as the word tokenization had only been added recently.

The improvement made by Thirion et al. consisted in limiting noise in MEDLINE and increasing recall in non-indexed PubMed subsets. When a MeSH term was used in a query, this improvement resulted in the retrieval of: 1) citations indexed with this same MeSH term in MEDLINE and 2) non-indexed citation containing any entry term for this MeSH term in its title or abstract. The corresponding query was: *q3 = 'preferred term"[MeSH term] OR (("preferred term"[TIAB] OR "entry term_1_"[TIAB] OR "entry term_2_"[TIAB] OR...) NOT Medline[SB]) *(Table 1). In contrast to the PubMed ATM, the strategy proposed by Thirion et al. provides the same query, whether or not the query includes preferred terms or entry terms.

In the current study, we propose a new strategy in order to increase recall: adding to the mapped queries all the UMLS synonyms with "ORs": *q4 = "preferred term"[MeSH term] OR (("preferred term"[TIAB] OR "entry term_1_"[TIAB] OR "entry term_2_"[TIAB] OR... OR "UMLS synonym_1_"[TIAB] OR "UMLS synonym_2_"[TIAB] OR...) NOT (Medline[SB] OR OldMedline[SB])) *(Table 1). The exclusion of OldMedline subset allows this query to focus on Pre-MEDLINE citations ("as supplied by publisher" and "in process" citations), which are not yet manually indexed by NLM curators. Non-indexed citations are not necessarily the latest citations. Nevertheless, according to the NLM customer service [8], time to index varies greatly between all of the different works that MEDLINE indexes. According to their recent statistical analysis, 25% of the citations are completed within 30 days of receipt, 50% within 60 days, and 75% within 90 days. Furthermore, 82% of Pre-MEDLINE citations that were evaluated in this study were published in 2011 and 11% in 2010. Obviously, when multiple UMLS synonyms contained the same spellings, they were not added in the mapped query. For technical purposes, we limited this list of synonyms to those included in the Health Multi-Terminology Portal http://pts.chu-rouen.fr[9]: SNOMED CT, SNOMED intl, ICD-10, WHO-ART, WHO-ICF, WHO-ICPC2, LOINC, MedDRA, FMA and MEDLINEPlus.

### Evaluation

For a quantitative assessment, the number of recent citations retrieved only by the new strategy was computed and compared to the entire number of recent citations retrieved.

To evaluate qualitative changes induced by this modification of mapping, we built Boolean queries based on MeSH terms: *q5 = q4 NOT q3 *(Table 1). We have selected 20 of the most frequently used MeSH Descriptors (according to the 2011 MEDLINE Baseline Repository data available at http://mbr.nlm.nih.gov/Download/index.shtml#MeSH) from the MeSH Diseases Category (C) where q5 provides citations. The choice of the C (diseases) tree from the MeSH thesaurus was driven by its potential impact on daily health care. Two medical librarians (BT and GK) and two physicians (LR and NG) assessed the relevance of the top 20 answers for each query manually after a careful reading of the title and abstract. Retrieved citations were assessed for relevance according to a three-modality scale used in other standard Information Retrieval test sets [10]: bad, partial or full relevance.

Three factors might have an impact on the number of citation retrieved: (a) the number of sons in MeSH hierarchy (b) number of MeSH synonyms (c) number of UMLS synonyms. These factors were recorded and any association was evaluated using Spearman's correlation.

Evaluators' agreement was measured using kappa statistics (SAS Macro MKAPPA [11]). Precision was computed at two levels of relevance: using only fully relevant or fully and partially relevant citation. They were then computed for each evaluator and compared using the Friedman test and Chi^2 ^test.

## Results

Table 2 summarizes results for the 43 queries we had to perform in order to obtain 20 citations for 20 queries. For the other 23 queries, q5 query did not produce any results: enhancing query using UMLS synonyms did not add any further results. The new strategy led to a heterogeneous 23.7% mean increase in non-indexed citation retrieved (from 0 to 9,876 new citations retrieved). None of the three tested factors (number of sons in MeSH hierarchy, of MeSH synonyms and of UMLS synonyms) were significantly correlated with the number of citations retrieved or the precision.

**Table 2:** Increase in non-indexed citation retrieved with the new method

Query Term	q5	Non-indexed citation with q3	Increase in non-indexed citation retrieved
Neoplasms	46	1871	2.5%

Hypertension	23	10547	0.2%

Myocardial infarction	155	5298	2.9%

Coronary disease	41	5397	0.8%

Asthma	133	4149	3.2%

Obesity	379	7552	5.0%

Liver neoplasms	641	266	241.0%

Diabetes Mellitus	9876	5033	196.2%

Inflammation	361	13019	2.8%

Heart Failure	272	6042	4.5%

Kidney Failure, Chronic	81	372	21.8%

Alcoholism	295	713	41.4%

Epilepsy	2470	3256	75.9%

Tuberculosis	1238	6752	18.3%

Liver cirrhosis	155	1983	7.8%

Kidney Diseases	2667	1095	243.6%

Cross Infection	167	1255	13.3%

Parkinson Disease	411	396	103.8%

Lymphoma	144	3939	3.7%

Hypersensitivity	159	1357	11.7%

Breast neoplasms	0	11	0.0%

Lung neoplasms	0	23	0.0%

Skin neoplasms	0	10	0.0%

Melanoma	0	2491	0.0%

HIV infections	0	142	0.0%

Brain Neoplasms	0	10	0.0%

Prostatic Neoplasms	0	0	-

Arthritis, Rheumatoid	0	0	-

Neoplasm Metastasis	0	0	-

Occupational Diseases	0	136	0.0%

Neoplasm Recurrence, Local	0	0	-

Substance-Related Disorders	0	25	0.0%

Pregnancy Complications	0	84	0.0%

Tuberculosis, Pulmonary	0	0	-

Genetic Predisposition to Disease	0	0	-

Wounds and Injuries	0	8	0.0%

Diabetes Mellitus, Type 1	0	1	0.0%

Ovarian Neoplasms	0	24	0.0%

Uterine Cervical Neoplasms	0	1	0.0%

Arrhythmias, Cardiac	0	0	-

Pancreatic Neoplasms	0	30	0.0%

Colorectal Neoplasms	0	14	0.0%

Lupus Erythematosus, Systemic	0	0	-

Total	19714	83341	23.7%

For the 20 studied MeSH Descriptors, inter-rater agreement was poor: multi-rater's kappa was 0.34. Results of relevance evaluation are summarized in Table 3. The mean precision for fully relevant citation was 48.4% CI_95% _= [45.8-50.9] but this number does not reflect discrepancies between evaluators: three evaluators (BT, GK and NG) found full relevance around 40% (43.7%, 36.7% and 37.4%, respectively) and one (LR) found 75%. Results are somewhat better for partially relevant citations but have a similar pattern, LR's evaluations were often more relevant than other evaluations: mean partial precision was 59.8% CI_95% _= [57.3-62.2]. BT, GK and NG found a precision of about 50% (50.1%, 51.7% and 48.2% respectively) whereas LR found 88.1%. Differences between evaluators were significant (p < 0.001, Friedman test). There was also a significant difference of precision depending on the MeSH term (data not shown, p < 0.001, Chi^2 ^test): for 8 MeSH term the full relevance precision was higher than 0.5, for 8 MeSH terms the partial and full relevance precision was less than 0.5.

**Table 3:** Precision, by experts and relevance

Expert	Precision [CI95%]	
	
	Partial and Full relevance	Full relevance
LR	88.1% [84.9-91.3]	75.0% [70.7-79.3]

NG	48.2% [43.3-53.2]	37.4% [32.6-42.1]

BT	50.1% [45.0-55.2]	43.7% [38.6-48.7]

GK	51.7% [46.7-56.7]	36.7% [31.8-41.5]

All	59.8% [57.3-62.2]	48.4% [45.8-50.9]

## Discussion

Enhancing information retrieval is one possible use of UMLS [12]. The new strategy led to a slight increase in non-indexed citation retrieval (23.7%) for a precision very similar to those observed in previous reports studying PubMed performances: Thirion et al. [7] showed a precision of 54.5%; Lu et al. [13], for a normal use of PubMed, found a mean rank precision for the 20 top results between 40% and 55%.

Nevertheless, this study has some limitations: First, the absence of a control group to make a comparison led to difficult interpretation of results. However, consistency with literature review suggests that there was no major bias. Second, we used queries based on one MeSH term from the "disease" tree (C). However, would the results be similar for other MeSH tree terms, queries including several MeSH terms or queries including MeSH terms and keywords? Third, there is great variation in the results of the expansion proposed here between queries. The three factors tested were not significantly correlated with precision, but a qualitative assessment of the results was manually performed:

(a) Some UMLS synonyms provide very good results (e.g. "hepatoma" for "liver neoplasms", "Nephropathy" for "Kidney Diseases"), probably because they are very similar to a son of MeSH Descriptor.

(b) Some UMLS synonyms are ambiguous acronyms that generate a lot of noise (e.g. TB for tuberculosis).

(c) Some MeSH descriptors correspond to frequent confounding factors (e.g. "hypertension", "obesity"). Results of retrieved citations are adjusted based on these factors but they are not the real subjects of the citations (mean precision for fully relevant citations: 21.1%, 23% respectively).

We have also tried to explain the number of newly retrieved citations using the q5 query, which varies from 0 to 9,876 (for Diabetes Mellitus; see Table 2).

(a) The number of UMLS synonyms greatly varies from 0 to 38, with a median of 10 (data not shown). This natural source of variation was not confirmed by correlation tests. The difference must be more qualitative:

(b) Some UMLS synonyms do not provide any added value in information retrieval (e.g. all the synonyms finishing by ", NOS" will not provide any citation).

Fourth, the relevance assessment was performed on title and abstract alone, but not with the full text of the article. Although this could have introduced a bias in this study, it seems to us more pragmatic as most end-users select the relevant citation based on title and abstract alone. Lastly, the poor inter-rater agreement measured here (kappa = 0.34) suggests that we do not really know what we are measuring, even if it is common for this type of study [14]. This poor kappa score, and the surprising distribution of results, only highlights differences between users. The improvement proposed here is probably not of interest for some users but may be of interest for others. Based on this study, we have implemented the following three procedures to query MEDLINE via PubMed in the following tool InfoRoute, French Infobutton (URL: inforoute.churouen.fr) [15]:

(a) The classical PubMed ATM

(b) The previous procedure developed by Thirion et al. (semantic expansion with MeSH Entry terms)

(c) The current procedure (semantic expansion with UMLS synonyms)

Different types of users should use these three procedures. Users expecting the most exhaustive results, even at the cost of some noise, should use the latest one. This type of users wants to maximize the recall.

Lu et al. [6] reviewed 28 different ways to access MEDLINE citations. The search strategy we propose could possibly be the 29^th^. However, when compared to other teams' strategy to improve PubMed information retrieval, the ones developed by our team modify the ATM and then are applicable in the PubMed interface. In fact, there is no need to integrate and update the MEDLINE bibliographic database in our information system.

Considering the huge number of citations retrieved by each q3 query (frequently more than dozens of thousands, data not shown), the increased number of recent citations retrieved may not lead to an important increase in recall. Nevertheless, the proposed strategy is based on the following assertion: a citation that is not indexed with a MeSH term does not have to be retrieved whatever the semantic expansion was used.

Based on this, the new strategy will only retrieve new citations not belonging to MEDLINE that represent more than ¾ of PubMed citation. We observed a 23.7% increase in recall for the citations aimed by the new strategy, which is not insignificant for the users, especially if they are searching for recent scientific advancements. This improvement mainly concerns new citations (82% of the citations retrieved by q5 have been published less than 4 months earlier). Furthermore, these citations, ranked first by PubMed, may be of great interest for PubMed users who frequently do not read more than the top 20 answers [16].

In contrast to PubMed, we assumed that when end users search for a disease name in PubMed, they do not add synonyms because of laxity or unawareness. It could be useful to add the son's preferred terms, son's entry terms and son's UMLS synonyms to the query with "ORs". This would eventually lead to an increase in recall and in proportion of queries retrieving additional citation (20 on 43 for this study) and a decrease in precision. However, it would drastically increase query size and resources needs, which are already quite substantial.

## Conclusions

The expansion of queries using UMLS' synonyms may not be of interest for all PubMed users, but could be quite useful when seeking for exhaustivity (review, meta-analysis, etc.) as well as when searching for the latest scientific citations. This study highlights the need for specific search tools for each type of user and use-cases.

## Abbreviations

ATM: Automatic term mapping; FMA: Foundational model for anatomy; ICD10: International classification of diseases: tenth revision; ICF: International Classification of Functioning: Disability and Health; ICPC2: International Classification of Primary Care: Second edition; LOINC: Logical Observation Identifiers Names and Codes; MedDRA: Medical Dictionary for Regulatory Activities; MeSH: Medical subject heading; NLM: National library of medicine; SB: Subset; SNOMED CT: Systematized nomenclature of medicine clinical terms; SNOMED intl: Systematized nomenclature of medicine international; TIAB: Title or abstract; UMLS: Unified medical language system; WHO: World health organization.

## Competing interests

The authors declare that they have no competing interests.

## Authors' contributions

JFG and SJD formulated the idea of this study, design it and participated in writing the draft. NG evaluated citations' relevance, made statistical analysis and wrote the draft. LR, GK and BT have evaluated citation's relevance and have participated in writing. WC has built queries, has retrieved results and has participated in writing the draft. All authors read and approved the final manuscript.

## Pre-publication history

The pre-publication history for this paper can be accessed here:

http://www.biomedcentral.com/1472-6947/12/12/prepub
